# Hints for Hospital Stewards

**Published:** 1911-09-09

**Authors:** 


### HINTS FOR HOSPITAL STEWARDS.

### KITCHEN IMPROVEMENTS AT THE BAKERS' EXHIBITION.

Public attention has of late been directed in an unusual degree to the subject of foodstuffs, especially bread, and this year the Confectioners' and Bakers' Exhibition at the Agricultural Hall will doubtless be more popular than ever. The scope of this exhibition includes a great variety of manufactures and trades, and among the firms showing there are many whose products are of considerable interest to managers of hospitals and large institutions. In the smaller hall the exhibit of Messrs. Schweitzer Bros. and Co., of 311 Oxford Street, is well worth a visit. This firm specialises in grinding machinery, and makes a feature of a complete installation for a milling bakery suitable for institutions and households. These installations can be had in graduated sizes according to the output desired, and comprise mill, sieve, kneader, and oven. The new French "Rational" mills of this system enable grinding to be carried out with great perfection and economy, and the flour produced by these mills is of a more nourishing quality than usual, and is therefore capable of being made into a very nutritive bread, approaching as nearly as can be a true healthy standard bread. The sieves used in the installations are of a most ingenious type, take up but little space, and are simple to keep in order, being automatically cleansed from any clogging. The whole of the necessary machinery for a complete milling bakery occupies quite a moderate amount of space, and should be a very desirable feature in the equipment of many institutions. The smallest size, suitable for a household, is priced at £18 complete. The Goodell Company, of 149 Queen Victoria Street, show a large variety of peeling and coring machines, which are widely used, and, indeed, indispensable in large kitchens where large quantities of potatoes and fruit have to be prepared daily. Some of the hand machines are exceedingly cheap, considering their efficiency. The storage of sugar, cereals, bread, salt, and such like is often not so carefully attended to as its importance demands, and difficulties arise through damp or deterioration. The presence of crevices allows of the lodgment of, for instance, flour, then the deterioration of this is liable to be communicated to fresh supplies. Messrs Young's hygienic storage bins fulfil the conditions demanded for an ideal receptacle. They are made of galvanised steel, slightly concave, so that no lodgment is offered for the contents, and if a bin be filled and then inverted the whole of the contents will be thrown out—an obviously important matter. The firm also make an excellent sugar bin, which keeps the cubes bright and clean and minimises the amount of sugar dust formed. All these bins are advantageously fitted to a neat trolley stand at a small extra charge, and their convenience is thereby much increased.

Mr. John B. Daniels is exhibiting a small size rustless dough-mixing machine, which is workable by hand, and has a capacity for one sack of flour. It is unusually cheap in price, and worth the attention of hospital stewards.

From bakehouses to black beetles is not a far cry, and it is not surprising to find that exhibits by firms dealing in vermin destroyers are to be found at the Agricultural Hall, and these may well receive passing notice here, seeing that hospitals are unfortunately by no means exempt from the attentions of vermin. The American Cockroach Solvent Co. claim to be the largest firm of vermin destroyers in the world, and undertake to clear premises of beetles and cockroaches on no-cure-no-pay conditions. This firm also has received considerable success in another direction—namely, in the destruction of rats, an object which, in the light of a recent epidemic, will have the benediction of the medical profession. The Ready Rat Relief virus has proved its efficacy on many occasions, and often on a large scale—for instance, on three of the London Tube Railways. Mr. B. L. Phillips explained many interesting observations and experiments in connection with the use and the preparation of the virus in the firm's laboratories, and pointed out that disease inflicted on rats was not only fatal in about seven to fourteen days, but was also often spread from animal to animal. The virus is, of course, a bacillary growth sent out in tubes of nutrient jelly, like bacteriological culture tubes, and is non-poisonous to man, fowls, domestic animals and cattle. The firm has a staff of 225 men engaged solely on the destruction of rats by virus, poison and trapping. It is to be hoped that hospital stewards have realised the many opportunities for securing improvements in their kitchens which this week's exhibition has afforded.

